# Shallow landslide disposition in burnt European beech (*Fagus sylvatica* L.) forests

**DOI:** 10.1038/s41598-019-45073-7

**Published:** 2019-06-14

**Authors:** Eric Gehring, Marco Conedera, Janet Maringer, Filippo Giadrossich, Enrico Guastini, Massimiliano Schwarz

**Affiliations:** 1 Bern University of Applied Sciences, Department of Agriculture Forestry, Food Science & Management, Langgasse 85, 3052 Zollikofen, Switzerland; 2 Swiss Federal Institute for Forest, Snow and Landscape Research WSL, Insubric Ecosystems Research Group, A Ramél 18, Cadenazzo, CH-6593 Switzerland; 3 University of Sassari, Department of Agriculture, viale Italia 39, 07100 Sassari, Italy; 4 University of Florence, Department of Agricultural, Food and Forestry Systems, Piazzale delle Cascine 18, 50144 Firenze, Italy

**Keywords:** Natural hazards, Plant sciences

## Abstract

Tree roots contribute significantly to soil strength on hillslopes. In the case of wildfires, this effect may abruptly vanish and be lacking for a considerable period of time depending on the resistance and resilience of the forest. Despite its importance, quantitative data on the impact and dynamics of wildfires on slope stabilization is still lacking. We use the study case of the *Fagus sylvatica* L. to quantify the medium-term evolution of root reinforcement and its effect on slope stability in fire-injured forests. In the study, we upscale root reinforcement using field data for the calibration of the Root Bundle Model and detailed information on forest structure in 244 plots, and calculate the spatio-temporal dynamics of forest protective capacity using a three-dimensional probabilistic slope stability model (slideforNET) for different site types. In unburnt and low-burn forests, the protective capacity was found to remain constant over time. Forests hit by moderate burns continue to provide adequate protection for shallow (depth < 0.5 m) and cohesive soils only, whereas in the case of high severity fires, the protective capacity vanishes for 15 years and an increased shallow landslide probability remains for at least 40 years. These conditions call for appropriate sylvicultural post-fire measures.

## Introduction

The protective function of forests against gravitational natural hazards in mountainous regions is one of their most important ecosystem services. These services help to maintain the habitability of mountainous regions^1,2^ and represent a highly effective way of implementing ecosystem-based solutions for disaster risk reduction (usually referred as Eco-DRR^3^). Forest management strategies aiming at ensuring the long-term effectiveness of such ecosystem services should be based on holistic and well-grounded scientific knowledge. For instance, forest stand species composition and structure potentially imply considerable differences in the resistance and resilience of forest systems against disturbances and the related protection against gravitational natural hazards^4^.

In the case of shallow landslides, it is well-known that predisposing factors mainly consist of soil cohesion, friction angle, slope gradient, and shear plane depth^5^. The presence of trees and their root systems, in particular, provide a significant increase in soil strength and slope stability^6–11^. Root reinforcement depends on the root distribution in the soil and their mechanical properties (e.g. strength under tension, compression and bending)^12–15^. However, roots are likely to be affected by the vegetation responses to soil properties^16,17^, climate^18–20^, and sudden disturbances such as timber harvesting^9,21–23^ or wildfire^24^. For example, tree death occurring after a stand-replacing wildfire also implies a subsequent progressive root decomposition process, which inevitably reduces the related soil stabilizing effect^24–26^. Consequently, depending on the disturbance and soil type, the stabilizing effect of a forest against shallow landslides may vary greatly in space and time^20,27–31^. However, the fire intensity-related medium-term dynamics in terms of root spatial distribution and root reinforcement have been neglected so far in literature. No quantitative information in particular exists on the post-fire evolution of the protection capacity of forest stands against shallow landslide, which should be a prerequisite for a precise and effective Eco-DRR approach.

In this work, we combine newly collected data on the root distribution and mechanical properties of European beech with the post-fire beech stand structure dataset provided by Maringer *et al*.^32^ to calculate the medium-term (i.e., 35 years) post-fire evolution of root reinforcement in mixed-severity (low, medium, and high) forest fires in the southwestern Alps. The overall aim of the study is to upscale root reinforcement quantification to the stand level for the purpose of modelling the spatio-temporal dynamics of forest protective function against shallow landslides. We define in this context the protection capacity of the forest as the percentage of landslides frequency reduced due to root reinforcement.

European beech (*Fagus sylvatica* L.) forests is used as a case study because of the reported increase in frequency and severity of natural disturbances in recent decades^33^, with particular emphases on fire^34–36^, what is common to most mountain forest species nowadays as it was reported for the Alps^37^. Beech is highly susceptible to forest fire due to its thin bark, which cannot protect the cambium from lethal heat release, and its age-dependent poor resprouting capacity^38–41^. Both traits may have, in turn, direct consequences on the protective capacity of the affected stand. Moreover, natural beech regeneration relies on seed dispersal by gravity and animals^42,43^ which are limited when fire intensity is high and mature trees become sparse^44^. Post-fire tree mortality occurs rapidly within the first years after high burn severity and is extended to within a 20-year period after moderate burn severity^45^. As reported by Maringer *et al*.^32^ such progressive mortality may result in a significant reduction in the forest protective capacity against rockfall between 10 and 30 years post-fire.

## Results

### Forest characteristics

From the 34 selected fire sites, plots classified as burnt (210 plots − 200 m^2^) and unburnt (34 plots near the burnt areas) were assessed. Data from the unburnt plots were merged with the low burn category (hereafter referred to as “low burn”) because of similarity of results also obtained by Maringer *et al*.^32^. This lead to 35% of the sampled plots being classified as high severity burn and the remaining two thirds as moderate (33%) and low (unburnt + low) severity (32%) burn. The average occurrence of beech was always higher than 80% in tree species composition. This was true for low and moderate severity burn plots, where coniferous (<=2%) and other broadleaved species (<=17%) rarely occurred. This differed for high severity burn plots, where beech reached barely 60% and broadleaved species reached up to 39% (Table 1).

**Table 1:** Forest characteristics by burn severity.

Forest characteristcs	Low burn	Moderate burn	High burn
Plot number (n)	77	81	86
Mean stand density (n/ha)	762	449	193
Tree number per DBH class (n)			
8–12 (cm)	136	93	101
12–24 (cm)	316	178	56
24–36 (cm)	182	105	26
>36 (cm)	128	73	10
Occurrence of dominant tree species (%):			
*Picea abies*	0	0	0
*Fagus sylvatica*	93	82	59
Other broadleaved species	6	17	39
Other coniferous species	2	1	2

Low burn class data includes unburnt plots.

**Figure 1:**
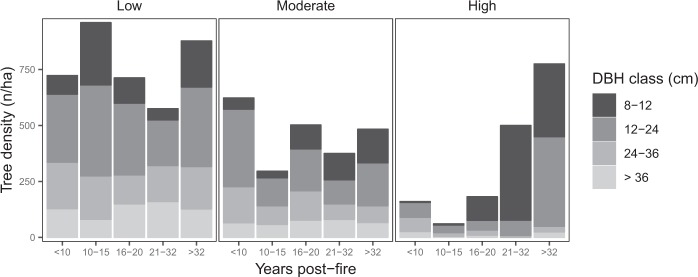
Living tree density (DBH ≥ 8 cm) by DBH class, burn severity, and years post-fire. Low burn class data includes unburnt plots.

The average mean stand density (number of living trees per hectare) was highest in the low burn plots (762 ha^−1^) and progressively decreased depending on burn severity up to 193 ha^−1^ in the high burn plots (Table 1). Specifically, the evolution of tree density by DBH class based on years since fire did not reveal any clear pattern in low and moderate burn plots (Fig. 1). In contrast, for high severity burns, also larger trees (24–36 cm and >36 cm DBH) are reduced in numbers, especially after 10 years post-fire. Contrariwise, the proportion of small-diameter trees (<12 cm) is initially (<16 years post-fire) very low and increases strongly from 16 years post-fire onwards becoming the most represented DBH class by far (Fig. 1 and Table 1).

### Post-fire root reinforcement dynamics

**Figure 2:**
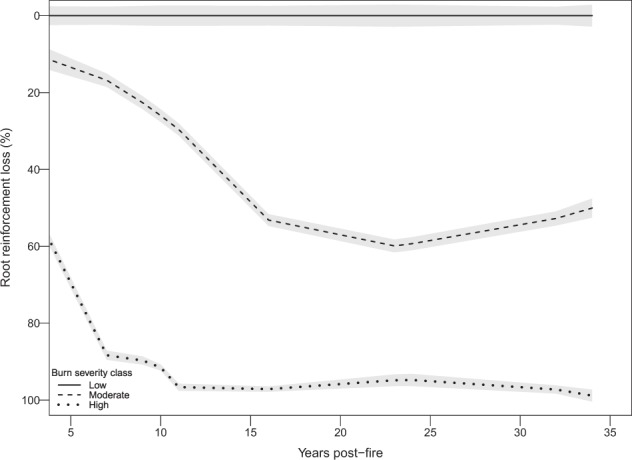
Evolution of post-fire root reinforcement loss by burn severity class. Low burn class data includes unburnt plots and serves as a reference class. Percentage of root reinforcement loss (rrl) is calculated as follow: (rrl_measured_ − rrl_reference_)/rrl_reference_ * 100. Lines represent the LOESS regression (span = 1), grey bands indicate the 95% confidence interval.

The evolution of post-fire root reinforcement shows similar yet distinct patterns depending on burn severity. In the case of moderate and high severity burns, ~15% and ~70% of root reinforcement is lost within the first 5 years post-fire, respectively. In moderate burns, the loss triples over the following years increasing to 60% by the 23^rd^ year post-fire (Fig. 2), whereas in high severity burn plots the loss doubles already within 10 years, reaching an almost total loss (98%) after 15 years post-fire. In both cases, no additional significant changes occur once the maximal loss is reached (Fig. 2).

### Forest protective capacity for different soils and scenarios

The mean forest protective capacity generally decreased with increasing slope and soil depth when considering burn severity classes separately (low-, moderate-, high-burn; Table 2). Comparing the protective capacity of low with moderate-/high-severity burns, significant decreases were found in all soil class and scenarios (Table 2).

**Table 2:** Mean protective capacity (%) with 95% confidence interval by soil class and different burn scenarios. The non-parametric Mann-Whitney U test with the Holm adjustment was used to identify significant differences between low burn (including unburnt) and moderate/high burn scenarios. Differences are significant at p-values as follows: ‘***’ 0.001; ‘**’ 0.01; ‘*’ 0.5; ‘·’ 0.1; ‘ns’ 1. NA indicates that, with these soil characteristics, generally no shallow landslides occur regardless of forest presence.

		Soil class								
		1			2			3		
Effective friction angle of the soil (deg)		25			25			35		
Effective soil cohesion (kPa)		0			2–5			0–2		
Mean slope		25°	35°	45°	25°	35°	45°	25°	35°	45°
Soil depth (m)	Burn severity	Mean protective capacity (%)								
0.5	Low	97 ± 3	62 ± 8	34 ± 8	NA	98 ± 3	88 ± 5	NA	95 ± 3	82 ± 5
	Moderate	82 ± 6***	28 ± 6***	9 ± 4***	NA	88 ± 6***	56 ± 8***	NA	76 ± 6***	59 ± 6***
	High	14 ± 6***	1 ± 2***	1 ± 1***	NA	17 ± 7***	5 ± 3***	NA	12 ± 6***	10 ± 4***
1	Low	72 ± 4	6 ± 2	0 ± 0	93 ± 3	65 ± 5	29 ± 5	NA	74 ± 3	24 ± 4
	Moderate	48 ± 5***	1 ± 1***	0 ± 0	76 ± 6***	47 ± 6***	13 ± 3***	NA	51 ± 4***	10 ± 2***
	High	6 ± 3***	0 ± 0***	0 ± 0	13 ± 6***	8 ± 3***	1 ± 1***	NA	8 ± 3***	1 ± 1***
1.5	Low	51 ± 4	1 ± 0	0 ± 0	75 ± 4	28 ± 4	1 ± 0	NA	50 ± 3	4 ± 1
	Moderate	31 ± 4***	0 ± 0***	0 ± 0	54 ± 5***	15 ± 3***	0 ± 0***	NA	31 ± 3***	2 ± 0***
	High	4 ± 2***	0 ± 0***	0 ± 0	8 ± 4***	2 ± 1***	0 ± 0	NA	4 ± 2***	0 ± 0***

**Figure 3:**
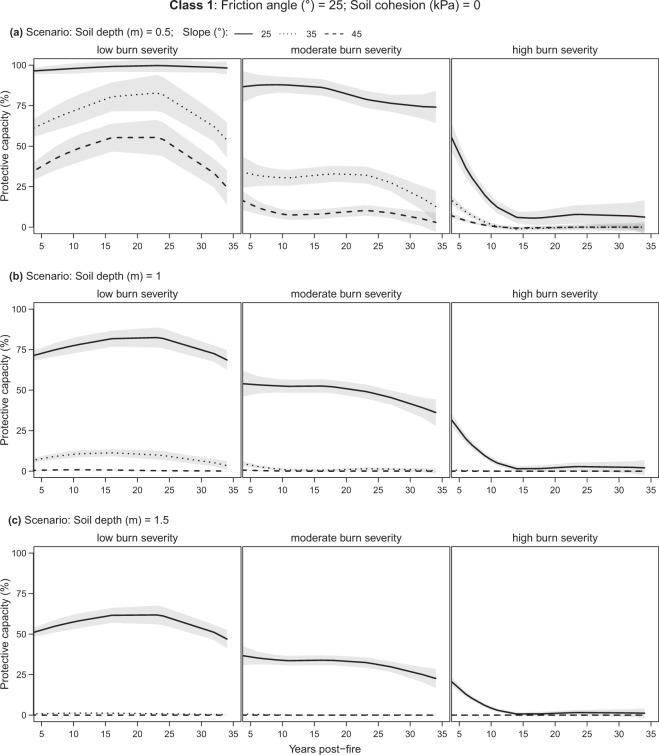
Evolution of the forest protective capacity against shallow landslides for soil class 1 by different fire severity classes, soil depths, and slopes. Soil *class 1*: friction angle = 25°, soil cohesion = 0 (kPa). Lines represent the LOESS regression (span = 1) and grey bands the 95% confidence interval.

**Figure 4:**
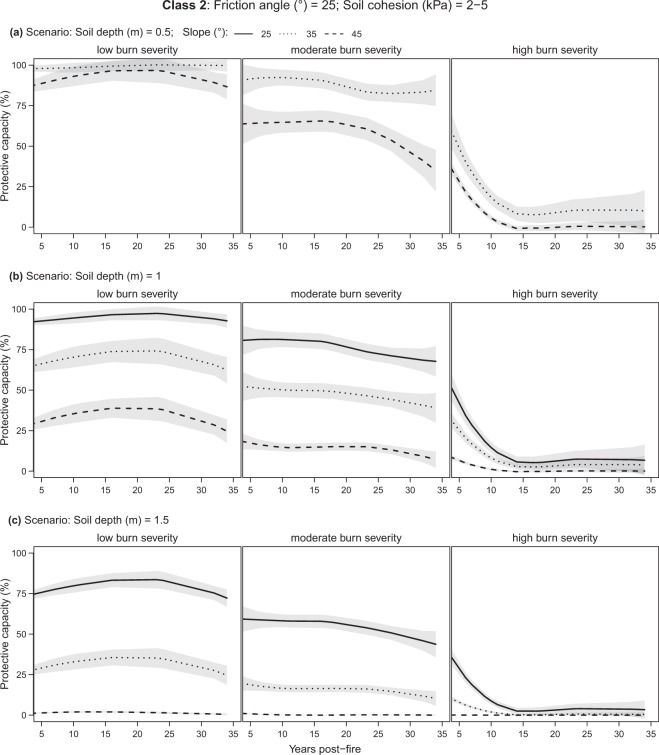
Evolution of forest protective capacity against shallow landslides for soil class 2 by different fire severity classes, soil depths, and slopes. Soil c*lass 2*: friction angle = 25°, soil cohesion = 2–5 (kPa). Lines represent the LOESS regression (span = 1) and grey bands the 95% confidence interval. Note that when slope <=25°, no shallow landslides occur and the forest protective capacity function does not apply.

**Figure 5:**
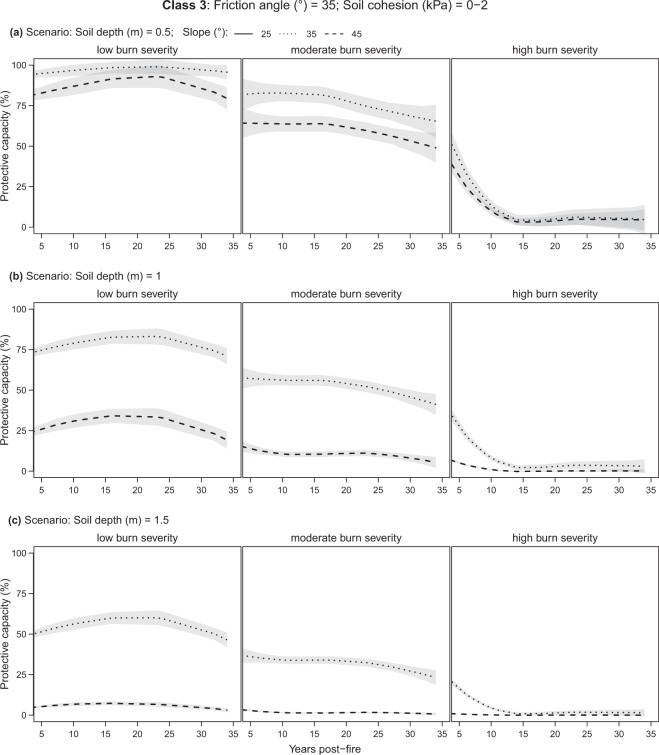
Evolution of forest protective capacity against shallow landslides for soil class 3 by different fire severity classes, soil depths, and slopes. Soil c*lass 3*: friction angle = 35°, soil cohesion = 0–2 (kPa). Lines represent the LOESS regression (span = 1) and grey bands the 95% confidence interval. Note that when slope <=25°, no shallow landslides occur and the forest protective capacity function does not apply.

Soil class 1 (non-cohesive soil) was found to consistently have the worst protective capacity conditions (Fig. 3) compared to classes 2 and 3 (cohesive soil; Fig. 4 and Fig. 5), decreasing as soon as the slope becomes steeper than 25° (Table 2). Soil class 3 is generally stable on gentle slopes (<=25°) regardless of forest presence or soil depth (NA in Table 2 and Fig. 5). Soil classes 2 and 3 behave similarly and provide a protective capacity of over 50% in low and moderate burns, as long as the soil is not deeper than one meter and the slope is not steeper than 35°. For steeper slopes (i.e., 45°), their protective capacity remains above 50% for shallow soils (<=0.5 m; Table 2) only. Finally, when burn severity is high, the protective capacity gets very low (<25%), regardless of the soil class or burn scenario.

### Temporal trends in forest protective capacity against shallow landslides

#### Low burn severity

In low burn forests protective capacity is generally constantly higher than 75% for shallow soils (<=0.5 m) and for soil classes 2 and 3, regardless of slope (Figs 4a and 5a). In contrast, for soil class 1 it decreases from high to medium-low as slope becomes steeper (Fig. 3a). For deeper soils (>0.5), it provides medium-high (>50%) protection on gentle slopes (25°) only (Fig. 3b,c). Soil classes 2 and 3 follow similar trends, although always with higher protection compared to class 1, becoming very low (<25%) for deep soils (1.5 m) with slopes of 45° (Figs 4c and 5c).

#### Moderate burn severity

In the case of moderate burns, all scenarios report lower protective capacity with respect to low burns with a slightly decreasing trend over the years (Figs 3, 4 and 5, moderate burn severity). After 35 years post-fire, protection capacity does not drop below 50% for shallow soils (0.5 m) and classes 2 and 3.

#### High burn severity

Finally, for high-severity burns, a protective capacity >50% is reached with burns less than six years post-fire and in shallow soils (0.5 m) only. Steeper slopes and deeper soils cause the protective capacity to drop below 50% for all soil classes. From six years post-fire onward, the protective capacity drastically decreases for all scenarios reaching minimum values (ca. <10%) at 15–20 years post-fire (Figs 3, 4 and 5, high burn severity).

## Discussion

In this study, we analyzed the medium-term temporal trends of the forest stabilizing effect in beech stands disturbed by forest fires of different severities. According to our simulations, even in unburnt or low severity burn forests the protection level against shallow landslides may be below 50%, especially in the case of deep soils (1.5 m) and steep slopes (45°).

Beech forests provide a good stabilizing effect for cohesionless soils (class 1) only when the slope is below 25° and/or the soil is not deeper than 0.5 m. As a general rule, as soon as the slope exceeds 35° and the soil is more than 1 m deep, protective capacity strongly decreases regardless of the soil type. Fortunately, these combinations of factors are rare, mainly because on steep slopes surface erosion strongly limits the accumulation of the soil mantle^46^. Low intensity wildfires cause little or no change in forest structure, including the amount and timing of regeneration^44^. This ensures a stable degree of protective capacity over the medium-term at a pre-fire level^32,47^.

In the case of moderate wildfires, delayed post-fire mortality due to secondary pathogens (i.e., fungi) and insects may, over the medium-term, gradually affect the protective capacity of the concerned stands, whereas incoming regeneration is still too young for significantly contribute to soil stability. The mean root reinforcement start to slightly recover after about 25 years, but beech stand dynamics after moderate fire severity are very heterogeneous^45^, so that the overall protective capacity tends to keep low. When high severity wildfires occur, forest structure changes abruptly causing a sudden decrease in protective capacity against shallow landslides, regardless of soil class, slope, or soil depth. According to Maringer *et al*.^29^, following high-severity burns, most intermediate and large beech trees die within the first ten years post-fire. Subsequently, root systems neither provide a soil-stabilizing function nor protection against shallow landslides. This is in line with other studies showing a total decay of the root system within a decade for beech forest^48^ and within 10/15 years for spruce (*Picea abies*^49,50^). Moreover, when severe wildfires occur, beech forests lose half of their protective capacity within the first five years post-fire due to the very high root decay rate, which may be as high as 11% per year^48^. In Scots pine (*Pinus sylvestris*) forests, root reinforcement has been found to decrease by a factor of 3.6 in four years post-fire^24^. Similarly, other studies highlighted a significant loss of strength after logging or clearcuttings that rapidly affected small roots (ca. two years after) and then progressively also larger ones (ca. ten years after)^23,51,52^. It is thus reasonable to assume that this will lead with time to an increased risk of landslide^25,31,49,53,54^.

The regeneration process of beech starts soon after the fire^44,55,56^ and ensures the future of beech forests. This is particularly true in the case of medium severity fires, where the gradual opening of the tree canopy due to the delayed mortality of injured trees creates suitable environmental conditions for beech recruitment and sapling growth^44^. In contrast, with respect to high severity burns, regeneration may be postponed because of the absence of seed providing trees or the temporary dominance of more competitive pioneer species that cause a momentary shift in the forest’s species composition^44^. Moreover, when such post-fire full light conditions occur under mild environmental conditions, pioneer alien woody species such as *Ailanthus altissima* and *Robinia pseudoacacia* may additionally invade large burnt areas, further limiting the regeneration process^41^. Over the long term (>32 years), this results in stands composed of a new generation of small- (DBH: <12 cm) and intermediate-sized (DBH: 12–24 cm) individuals (beech and/or pioneer trees) combined with large-sized (>36 cm) pre-fire surviving beeches^44^. This particular forest structure appears to provide adequate protection against rock fall^32^, although corresponding root systems are not yet sufficiently developed to provide adequate stabilization for shallow landslide-prone slopes. In contrast to previous studies^33,51,53–55^, our approach allow to consider the effects of both lateral and basal root reinforcements^44^ on shallow landslides of different sizes. In order to generalizes the results, the distribution of the considered landslide sizes is statistically representative for the Alpine regions^44^ whereas the chosen values ranges of the soil mechanical parameters refer to most of the real conditions. Furthermore, the simplification of the hydraulic conditions by assuming full saturated soils, allow a conservative comparison of the temporal dynamic of protective capacity of forests.

## Conclusions

In the present study, we reconstruct for the first time the quantitative medium-term evolution of the protection effects of European beech forests in terms of shallow landslide reduced probability considering a broad combination of factors such burn intensity, soil types, slope inclination and landslide depth.

Our results show that burnt beech forests that experienced low-severity fires does not show any post-fire decreasing trend for the whole study period (i.e., 37 years). In the case of moderate to high severity fires, the protection function of the forest completely vanishes within 15 years, without any sign of medium-term recovery even though regeneration usually starts soon after the fire event. Related shallow landslide probability likely remains high for 40–50 years post-fire.

In order to implement effective Ecosystem-based solutions for Disaster Risk Reduction (Eco-DRR) it is thus highly important to properly plan sylvicultural and/or technical measures in areas where the risk for humans and/or infrastructures is high. The proposed approach for quantifying the temporal dynamic of the post-fire root reinforcement can be easily extended to other disturbances (e.g. windthrow) and other disturbance-sensitive forest species. Most sensitive and priority areas for applying such approach may be easily identified by combining disturbance severity with local geomorphological (elevation, slope, and micro-topography) and forest (stand composition and structure) parameters. This approach may gain importance in future in view of ongoing climate change and increasing population and infrastructure densities in mountainous regions.

## Methods

### Methodological workflow

The quantification of forest protective capacity is calculated by combining different sets of input parameters that describe the distribution and mechanical properties of roots as well as the post-fire stand dynamics of European beech forests. In the first step, the acquired field data on root distribution and root pullout tests^57^ are used to calibrate the analytical model for the estimation of maximum root reinforcement as a function of tree dimension (DBH), distance from tree, and soil depth^4^, hereafter referred to as “root reinforcement dataset”. Subsequently, the data on post-fire beech stand structure (i.e., number of stems and related DBH, hereafter called “forest dataset”)^32^ is used to upscale the root reinforcement estimation at the stand scale^58^. Finally, different combinations of soil mechanical conditions, landslide depth, and slope inclination are considered to simulate the post-fire forest protective capacity using the 3-dimensional probabilistic slope stability model SlideforNET^47,58^ (see “Slope stability calculations and forest protective capacity“ subchapter for details).

### Root reinforcement dataset: upscaling approach for root reinforcement

The root reinforcement dataset was collected in four different study sites (Schangnau, Spissibach, Laura, Bremgarten) as described in Table 3.

**Table 3:** Study site characteristics for the root reinforcement dataset.

Site	Coordinate	Altitude [m.a.s.l.]	Exposition	Geology	Soil (WRB)	N° Soil trenches
Schangnau	46°48′56.6″ N 7°49′23.6″ E	1000	N	Sandstone	Umbrisols	4
Spissibach	46°38′12.739″ N 7°46′33.730″ E	1240	N-W	“Flysch”	Umbrisols /Stagnosols	6
Laura	46°12′42.822″ N 9°06′04.627″ E	1370	S-E	Sandstone	Umbrisols	16
Bremgarten	47°20′43.258″ N 8°19′52.813″ E	425	S	Sandstone	Umbrisols	10

Field root pullout tests and root distribution data were used to quantify the maximum tensile force of root bundles (Root Bundle Model - RBMw^59^) and for calculating the overall root reinforcement within the whole root system^4^.

The RBMw parameters were calibrated for beech using so far unpublished own data from 22 field pullout tests on coarse roots with diameters of up to 2.5 cm, and data on 191 labor tensile tests with root diameters of up to 6 mm available in literature^60^. Field tests were performed following the method described in Vergani *et al*.^61^, whereas the procedure for the tensile tests are described in Bischetti *et al*.^60^. The equations fitted for the RBMw are described in detail in Dazio *et al*.^4^. The maximum pullout force *F*_*max*_ as a function of root diameter, *ϕ*[*m*], is calculated as

1 $${F}_{max}(\varphi )={F}_{0}{\varphi }^{\alpha }\frac{1}{2}[1+erf(\frac{\varphi -{\varphi }_{m}}{{\varphi }_{sd}\sqrt{2}})]\,[N]$$

where *F*_0_ = 909,501[*N*], *α* = 1.43[−], *ϕ*_*m*_ = 0.0031[*m*], and *ϕ*_*sd*_ = 0.0023[m] are the values of the fitted coefficients.

The survival function used to calculate the progressive failure of roots due to their mechanical variability is

2 $$S({\rm{\Delta }}{x}^{\ast })=exp[\,-\,{({\rm{\Delta }}{x}^{\ast })}^{\omega }]\,[\,-\,]$$

where Δx^*^is the normalized pullout displacement and *ω* = 2.24[−].

The calculated values of maximum root reinforcement for each soil trench, where root distribution was measured, were used to calibrate the equation parameters used to estimate the root reinforcement, *RR*_*max*_[*Nm*^−1^], as a function of tree dimension, *DBH*[*m*], and distance from the tree stem, *d*[*m*], as follows:

3 $$R{R}_{max}(DBH,d)=\{\begin{array}{ll}a\,DBH\,{\rm{\Gamma }}(\frac{d}{DBH\,18.5},\,b,\,c), & for\,d < DBH\,18.5\\ 0, & for\,d\ge DBH\,18.5\end{array}$$

where Γ is the gamma density function, *a* = 25,068.54[*Nm*^−1^], *b* = 0.862, and *c* = 3.225. For determining the coefficient of 18.5, please refer to Schwarz *et al*.^62^.

The vertical distribution of root reinforcement, *RR*_*basal*_[*Pa*], is calculated using the equation

4 $$R{R}_{basal}(z)={{\rm{RR}}}_{max}{\rm{\Gamma }}({\rm{z}},{{\rm{z}}}_{\alpha },{{\rm{z}}}_{\beta })$$

where *z*_*α*_ = 1.284 and *z*_*β*_ = 3.688 are the calibrated coefficients of the gamma density function, and *z* is the soil depth.

Equations 3 and 4 are used to calculate the minimum values of basal and lateral root reinforcement at stand scale considering the forest structure dataset, as described in Dazio *et al*.^4^. In contrast to other studies^33,51,53–55^ dealing with the decay-related progressive decrease of the root reinforcement, this study focus on the dynamic of root reinforcement as determined by the fire-surviving trees.

### Forest dataset

The forest dataset refers to European beech forest stands in the southwestern European Alps across Canton Ticino (Switzerland) and Piedmont (Italy), where the species is usually concentrated at an intermediate elevation belt ranging from 600 to 1,700 m a.s.l. and represents 16% in Switzerland and 26% in Italy, respectively of the protection forests^63,64^. The species is a deciduous broadleaf tree with a cordate root system^65^ characterized by an intensive development of fine roots in the upper soil layer^66^ directly related to various factors such as climate, age, BHD, and stand composition^66–69^. Study design is described in detail in Maringer *et al*.^28^, and basically consisted in selecting beech–dominated stands (>95% of the stems) that experienced a single fire event of at least 0.25 ha in size between 1970 and 2012 and did not experience any anthropogenic activity (salvage logging, afforestation, pasture) since the fire event. Spanning the selection over 40 years allowed us to use a space-for-time approach to model the long-term dynamic of the post-fire protection capacity against shallow landslides.

Due to the high relief energy in the study region, which fosters heterogeneous burns, single sample plots where additionally categorized according to the resulting burn severity (low, moderate, and high) using the proportion of tree crown volume and basal area losses with respect to the pre-fire conditions as a fire severity proxy. For a thorough description of field data collection methodology, please refer to Maringer *et al*.^32^.

### Slope stability calculations and forest protective capacity

#### SlideforNET

The web tool SlideforNET (www.slidefor.net) bases on a probabilistic approach that considers the landslide size probability distribution. It enables the estimation of the contribution of root reinforcement to slope stability and the comparison of the related protective effect based on different forest types and slope. Details on the model calculation are presented in Schwarz *et al*.^47,58^. In SlideforNET, the slope stability calculation refers to a 3D force balance that assumes shallow landslides to be elliptical in shape, and provides a real-event-based probabilistic estimation of the landslide dimension^46,70^. Root reinforcement is then calculated by taking into account 1) the roots crossing the upper margin of the landslide (lateral root reinforcement along the potential tension crack) and 2) the roots crossing the basal shearing plane (basal root reinforcement, Eq. 4). The number of calculated landslides represents the partial probability that they may occur within a certain area under fully saturated conditions and that they are not influenced by a specific rainfall event magnitude or return period. The input parameter (shearing plane depth) as well as effective soil shear strength parameters (soil friction angle and soil cohesion) are defined based on scenarios covering all plausible conditions where shallow landslides may occur. For each scenario, 10,000 slope stability calculations with different randomly generated landslide dimension^46,70^ are computed.

We in particular considered three classes of soil mechanical properties obtained by grouping soils with similar friction angle (deg) and soil cohesion (kPa) based on the Unified Soil Classification System (USCS)^71^ as follow: class 1 (non cohesive soil) = 25 deg and 0 kPa; class 2 (cohesive soil) = 25 deg and 2–5 Kpa, class 3 (cohesive soil) = 35 deg and 0–2 kpa. The three classes retained for landslide depth failure were 0.5, 1, and 1.5 m, whereas the three classes of mean slope inclination were 25°, 35°, and 45°, respectively.

Different fire-injured beech forests conditions (unburnt + low-, moderate-, high-burn) were considered in the modeling in order to assess post-fire beech forest protective capacity against shallow landslides. The resulting forest protective capacity expressed as the percentage of landslides frequency reduced due to root reinforcement is then grouped into the following categories: ≥90% - very high protection against shallow landslides, 75–90% - high, 50–75% - medium-high, 25–50% - medium-low, and <25% - very low protection.

In order to check the robustness of the slideforNET model, two sets of simulations were run. Results showed that no significant difference in forest protective capacity values were found in any scenario between the two simulation sets, using a nonparametric Wilcoxon signed-rank test with the Holm adjustment for *p-*values. Overall, maximum differences ranged from −7.8 to 10.8% with only 1.2% (21 cases out of 1,772) greater than 5%.

### Statistical analysis

In order to consider the combination of factors that are representative for shallow landslide exposed slopes only, we limited the analysis to scenarios (i.e., combination of soil class, slope, and soil depth failures) with more than 1% probability that such events may occur (i.e., where at least 100 out of the 10,000 potential calculated landslides resulted unstable when excluding the effect of vegetation. Moreover, due to the lack of data on different fire severity scenarios, only sites whose fire event was more recent than 41 years ago were retained for the analysis.

Finally, due to the lack of significant impact of low severity fires on the beech stand composition and density^32^, unburnt and low severity plots were grouped in a single category.

Post-fire temporal trends in forest protective capacity were reconstructed using LOESS regression (locally estimated scatterplot smoothing^72,73^ due to the nonlinear response. The LOESS span parameter that determines the proportion of points in the plot which influence the trend at each value, was set to 1 in order to increase smoothness and thus visualize general trends^74^. Possible significant differences in the protective capacity among the different burn classes (unburnt + low, moderate, and high severity) were tested by means of a univariate comparative analysis using a Mann-Whitney U test.

All analyses were performed using the R software (version 3.4.1; R Core Team 2017). The main additional packages used were ggplot2 for producing graphics^75^, dplyr^76^ and tidyr^77^.

## Data Availability

The datasets generated during and/or analyzed during the current study are available from the corresponding author on reasonable request.
